# Selective Vulnerability of the Cochlear Basal Turn to Acrylonitrile and Noise

**DOI:** 10.1155/2009/908596

**Published:** 2009-05-06

**Authors:** B. Pouyatos, C. A. Gearhart, A. Nelson-Miller, S. Fulton, L. D. Fechter

**Affiliations:** ^1^Research Service, Jerry Pettis Memorial Veterans Affairs Medical Center, Loma Linda, CA 92357, USA; ^2^Centre de Recherche du Service de Santé des Armées (CRSSA), 24 Avenue des maquis du Grésivaudan, 38702 La Tronche, France

## Abstract

Exposure to acrylonitrile, a high-production industrial chemical, can promote noise-induced hearing loss (NIHL) in the rat even though this agent does not itself produce permanent hearing loss. The mechanism by which acrylonitrile promotes NIHL includes oxidative stress as antioxidant drugs can partially protect the cochlea from acrylonitrile + noise. Acrylonitrile depletes glutathione levels while noise can increase the formation of reactive oxygen species. It was previously noted that the high-frequency or basal turn of the cochlea was particularly vulnerable to the combined effects of acrylonitrile and noise when the octave band noise (OBN) was centered at 8 kHz. Normally, such a noise would be expected to yield damage at a more apical region of the cochlea. The present study was designed to determine whether the basal cochlea is selectively sensitive to acrylonitrile or whether, by adjusting the frequency of the noise band, it would be possible to control the region of the auditory impairment. Rats were exposed to one of three different OBNs centered at different frequencies (4 kHz, 110 dB and 8 or 16 kHz at 97 dB) for 5 days, with and without administration of acrylonitrile (50 mg/kg/day). The noise was set to cause limited NIHL by itself. Auditory function was monitored by recording distortion products, by compound action potentials, and by performing cochlear histology. While the ACN-only and noise-only exposures induced no or little permanent auditory loss, the three exposures to acrylonitrile + noise produced similar auditory and cochlear impairments above 16 kHz, despite the fact that the noise exposures covered 2 octaves. These observations show that the basal cochlea is much more sensitive to acrylonitrile + noise than the apical partition. They provide an initial basis for distinguishing the pattern of cochlear injury that results from noise exposure from that which occurs due to the combined effects of noise and a chemical contaminant.

## 1. Introduction

Acrylonitrile (ACN; vinyl cyanide) is an industrial chemical used extensively in the plastic, butyl rubber, and textile industries (SRI 1984). Approximately 125 000 workers are exposed to ACN daily in the US [1]. While the maximum permissible exposure level to ACN is quite low (1 ppm), exposure can quickly reach levels that exceed 1 ppm via skin contact in case of accidental exposure [2].

In laboratory animal models, high level exposure to acrylonitrile does not cause permanent auditory damage by itself [3–5], but can increase the vulnerability of the cochlea to moderate noise. These studies provide a means for testing the mechanisms responsible for potentiation of NIHL by chemicals rather than a direct model for occupational exposure. Pouyatos et al. [4] demonstrated that, when subjected to a combined exposure to ACN and to a 97 dB octave band noise centered at 8 kHz (OBN-8 kHz), rats displayed large permanent distortion product otoacoustic emissions (DPOAEs) deficits and compound action potential (CAP) threshold shifts along with marked outer hair cell (OHC) loss. The same exposure to noise alone did not yield any damage. Functional impairment was characterized by large high-frequency hearing loss (30–35 dB above 16 kHz based upon DPOAE and between 20–25 dB based upon CAP threshold), accompanied by a near total disappearance of the cochlear basal turn's OHCs. Because the cochlea is organized in a tonotopic manner with high frequencies encoded at the base and lower frequencies encoded at more apical locations in addition to the base, this type of noise by itself should have given a maximum hearing loss and cochlear damage centered on the middle turn at a location where tones of 12 kHz are encoded according to the half octave shift rule postulated by McFadden [6]. This study was designed to characterize further the role of noise and ACN exposure in auditory impairment that results from the combined exposure to these agents. This study also aimed to identify disparities between noise energy bands in the environment and physiological impairments that may be useful in estimating the role that chemical contaminants might play in hearing loss when noise is also present, for example, in occupational settings.

## 2. Materials and Methods

### 2.1. Subjects

A total of 57 male Long-Evans rats (225–249 g, 7–8 weeks old) obtained from Harlan (Indianapolis, Ind, USA) were employed in these experiments. The subjects were housed with free access to food and water in their home cages. Temperature was maintained at 21 ± 1°C and lights were on from 6 : 30 am to 6 : 30 pm. The Loma Linda Veteran Medical Center Institutional Animal Care and Use Committee (IACUC) approved all the experimental protocols. All exposures and testing were performed during the daytime.

### 2.2. Procedures

Groups of Long-Evans rats (n = 4–9) were exposed for 5 days (4 hours/day) to moderately intense octave band noise (OBN) centered at 4 kHz (110 dB SPL), 8 kHz (97 dB SPL), or 16 kHz (97 dB SPL), with or without co-exposure to ACN. The resultant permanent auditory impairments were assessed by evaluating loss of DPOAE amplitude within subjects between a pre-exposure measurement and assessment 4 weeks after exposure ended. Additionally, auditory thresholds were measured 4 weeks postexposure using the CAP. Cochlear damage was quantified in the same animals by systematic hair cell counts.

Experimental groups and exposure schedule are detailed in Table 1.

Control (*n* = 15) and ACN alone (*n* = 3) animals were maintained for 4 hours daily in the exposure chambers with the noise generator turned off. Due to technical reasons, DPOAEs were not recorded in 3 rats that received OBN-16 kHz alone and in 2 rats that received ACN + OBN-16 kHz. Control and noise-alone animals received saline injections in place of ACN.

### 2.3. Acrylonitrile Exposure

Stabilized ACN (99%) was purchased from Sigma-Aldrich (St. Louis, Mo, USA). ACN (50 mg/kg) injections were made daily for 5 days, 30 minutes prior to the noise. Fechter et al. [3] showed that ACN caused slight transient hearing impairment that reach a maximum at 10–20 minutes and resolve by about 75–100 minutes. The interval between ACN injection and onset of noise was selected based upon this known ACN-induced temporary threshold deficits.

### 2.4. Noise Exposure

**Figure 1:**
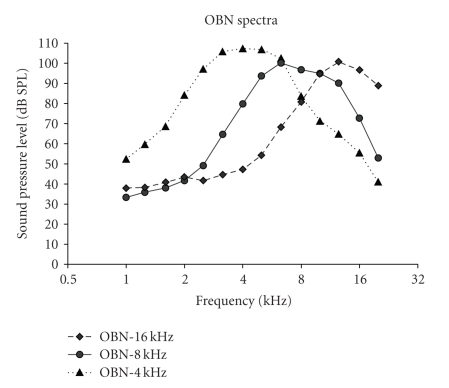
Noise spectra of the 110-dB-SPL octave-band centered at 4 kHz, and the 97-dB-SPL octave-bands centered at 8 kHz and 16 kHz.

Exposures were conducted in a ventilated reverberant 40 L Plexiglas cylinder. The subjects were placed within small wire-cloth enclosures (20 × 9 × 15 cm) within the chamber. They were conscious and free to move within the enclosures. Broadband noises were generated by a function-generator (Stanford Research System, Model DS335, Menlo Park, Calif, USA) and bandpass filtered (Frequency Devices, 9002, Haverhill, Mass, USA) to provide OBN with center frequencies of 4, 8 or 16 kHz. The roll-off for the filter system was 48 dB/octave. This signal was amplified by a SAE 2200 Power Amplifier (Scientific Audio Electronics Inc., Los Angeles, Calif, USA) and fed to speakers (Vifa D25AG-05, Videbaek, Denmark) located approximately 5 cm above the subjects' wire-cloth enclosure. Sound intensities measured at the level of the rats' pinnae by a Quest Type 1 sound pressure meter with 1/1 octave filter set (models 1700 and OB300, Oconomowoc, Wisc, USA) were 110 dB SPL for the OBN-4 kHz, and 97 dB SPL for the OBN-16 kHz and the OBN-8 kHz (Figure 1). The noise level chosen for the 4 kHz-OBN was 13 dB higher than the two other OBNs in order to compensate for the rat's higher auditory thresholds at low frequencies. Empirically, the rat's CAP threshold as determined in this laboratory is around 35 dB SPL in the octave band centered at 4 kHz, relative to 20 dB SPL for the 8-kHz and the 16-kHz OBNs. The noise spectra are displayed in Figure 1. Noise exposures lasted 4 hours for 5 successive days. The noise varied less than 2 dB within the exposure chamber.

### 2.5. Distortion Product Otoacoustic Emissions (DPOAEs) Testing

The rats were lightly anesthetized by injection of xylazine (7 mg/kg *im*) and ketamine (44 mg/kg *im*), and placed on a heating table in order to maintain the body temperature at 38°C. An Etymotics Research probe (ER10) was inserted in the right auditory canal in order to deliver the primary tones to the ear canal and record the DPOAE response. The same ear was subsequently used for CAP determination.

The primary tones, *F1* and *F2*, were generated by a dual-channel synthesizer (Hewlett Packard Model 3326A) and attenuated, under computer control, using customized software. The *F1* and *F2* primaries were then presented through two separate earphones (Radio Shack, Realistic Dual Radial Horn Tweeters, Tandy Corp., Ft. Worth, Tex, USA) and delivered to the outer-ear canal through a probe, where they acoustically mixed to avoid artifactual distortion. Ear-canal sound pressure levels, measured by an emissions microphone assembly (Etymotic Research, ER-10B+, Elk Grove Village, Ill, USA) embedded in the probe, were sampled, synchronously averaged, and Fourier analyzed for geometric mean (GM) frequencies ((*F*1×*F*2)^0.5^) ranging from 5.6 to 19.7 kHz (i.e., *F2* = 6.3–22.5 kHz) by a computer-based DSP board. Corresponding noise floors were computed by averaging the levels of the ear-canal sound pressure for five frequency bins above and below the DPOAE frequency bin (±54 Hz).

For test frequencies above 20.1 kHz, a computer-controlled dynamic-signal analyzer (Hewlett Packard Model 3561A) was used. The related noise floors were estimated by averaging the levels of the ear-canal sound pressure for the two FFT frequency bins below the DPOAE frequency (i.e., for 3.75 Hz below the DPOAE). No artifactual DPOAEs were ever measured in a hard-walled cavity that approximated the size of the rat outer-ear canal, which was used to calibrate the tonal stimuli. DPOAEs were considered to be present when they were at least 3 dB above the noise floor. DPOAEs were measured as DP-grams. Specifically, DP-grams described emission levels in response to primary tones at *L* *1* = *L2* = 75 dB SPL as a function of the GM frequencies, which ranged from 2.9 to 56.3 kHz (*F*2 = 3.2 to 63 kHz), in 0.1-octave increments. The ratio *F*2/*F*1 was 1.25.

Between 20 and 25 kHz (*F*2 = 20.8, 22.2, 23.8, and 25.6 kHz), the DP-grams display an artifactual notch due to the resonance of the rat's outer auditory meatus. Therefore, these frequencies were excluded from statistical analysis. Candreia et al. [7] previously observed a similar notch in the mouse. This phenomenon was recently described in detail by Martin et al. [8].

### 2.6. Compound Action Potentials (CAPs)

CAP threshold assessment was performed 4 weeks postexposure, the day after the last DPOAE measure. CAPs recorded from the right round window were elicited with pure tones bursts at 2, 4, 6, 8, 12, 16, 20, 24, 30, 35, and 40 kHz. The test tones were presented using a 1/2” ACO Pacific (Belmont, Calif, USA) model 7013 condenser microphone that was driven by a high impedence amplifier. The microphone was held in a plastic speculum within the ear canal. The stimuli were 10 msec in duration with a 1 msec onset and offset ramp. Stimuli were presented at a frequency of 9.7 Hz. Auditory thresholds were assessed in a double walled sound booth. The subjects were anaesthetized with xylazine (13 mg/kg *im*) and ketamine (87 mg/kg *im*) and normal body temperature was maintained using a heating table. The temperature of the cochlea was maintained using a low voltage high-intensity lamp. The auditory bulla was opened via a ventrolateral approach to allow the placement of a fine (od 0.1 mm) Teflon-coated silver wire electrode (A-M system, Inc., Carlsborg, Wash, USA) onto the round window. A silver chloride reference electrode was inserted into neck musculature. The CAP signals evoked by pure tones were amplified 1000x between 0.1–1.0 kHz with a Grass A.C. preamplifier (Model P15, W. Warwick, RI, USA). The sound level necessary to generate a visually detectable CAP response averaged over 4 sweeps on a digital oscilloscope (approximate response amplitude of 1 mV at the output of the preamplifier) was identified.

### 2.7. Hair Cell Counts

Immediately after CAP measurements, rats were decapitated and the cochleae harvested. Within 2 minutes, the cochleae were fixed by perilymphatic perfusion with 1 mL of a trialdehyde fixative (3% glutaraldehyde, 2% formaldehyde, 1% acrolein and 2.5% DMSO in phosphate buffered saline pH7.4). Following the primary 24-hour fixation, the tissue was first washed with 0.1 M phosphate buffered saline, post-fixed with 2% osmium tetroxide in water for 2 hours, and finally washed again with 0.1 M phosphate buffered saline. The organ of Corti was dissected in 70% ethanol and mounted in glycerin to allow counting of the hair cells using a surface preparation. Cells were counted as present either when the stereocilia, the cuticular plate or the cell nucleus could be visualized. No attempt was made to assess the degree of possible cellular damage to surviving cells. The frequency-place map established by Muller [9] was used to superimpose the frequency coordinates on the length coordinates of the organ of Corti. This “map” reflects the fact that the cochlea is organized in a tonotopic fashion with high frequency sound producing maximum stimulation of cells in the base, and low frequency sound in the apex. A cochleogram showing the percentage of hair cell loss as a function of distance from the apex of the cochlea was plotted for each animal. The results were averaged within each group of subjects for comparison between groups. The custom programs used for counting cochlear hair cells, plotting, and averaging cochleograms, were developed by R. Lataye and Dr. P. Campo from the “Institut National de Recherche et Sécurité,” Nancy, France. In some instances, the most basal region of the cochlea was damaged or not recovered during dissection owing to the fragility of the organ of Corti and difficulties extracting the hook at the extreme base from the surrounding bone. The degree of loss was estimated as 10 hair cells in length. In such cases all cells were considered to be present. Because such difficulties do not affect the apical region of the cochlea, cell counts were made from the apex of the cochlea to the base. Thus is was possible to be confident of the relationship between damage along the organ of Corti and the corresponding tone frequencies encoded at that locus.

### 2.8. Statistical Analysis

All statistical analyses were conducted using Prism 4.0c (GraphPad Software, San Diego, Calif, USA).

DPOAE amplitudes were analyzed with a global 2-way ANOVA regrouping all experimental groups using “exposure” as a between-subject factor and “*F*2 frequency” as within-subject factors. The 4 frequencies corresponding to the notch in the DPgram, (*F*2 = 20.8, 22.2, 23.8, and 25.6 kHz) were excluded from the analyses. Planned post hoc comparisons were performed between treatment groups using the Bonferroni's test. *P* = .05 was considered as the significance threshold.

Similarly, CAP thresholds were analyzed with a global two-way ANOVA to evaluate the effects of “exposure” (between-subject factor) at the different “frequencies” (within-subject).

OHC loss obtained in all experimental groups was analyzed conjointly with a global two-way ANOVA with the “exposure” as a between-subject factor and “cochlear location” of the hair cell loss as a within-subject factor. Bonferroni's post-hoc tests were performed between experimental groups. *P* = .05 was considered as the significance threshold.

## 3. Results

### 3.1. Functional Data

#### 3.1.1. Distortion Product Otoacoustic Emissions

**Figure 2:**
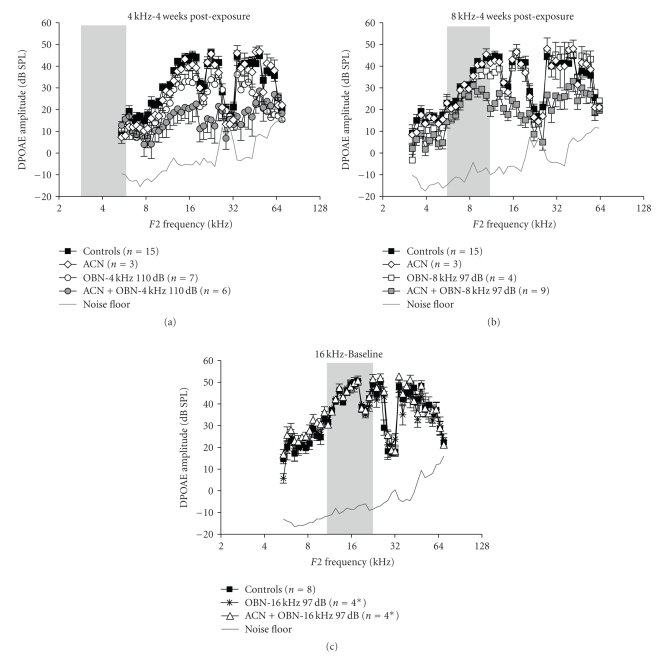
Four-week postexposure DPOAE amplitudes obtained from rats exposed to (a) the 110 dB OBN-4 kHz with or without ACN, (b) to the 97 dB OBN-8 kHz with or without ACN, or (c) to the 97 dB OBN-16 kHz with or without ACN (see Table 1 for details). DPOAE amplitude measured in control and ACN alone animals were included in each of the graphs for reference. DPgrams were obtained with the levels of the primaries *F*1 and *F*2 set at 75, and with *F*2/*F*1 = 1.25. The tested *F*2 frequencies ranged from 3.2 to 63 kHz (geometric mean frequencies: 2.9 to 56.3 kHz), in 0.1 octave increments. The gray areas represent the theoretical octave-band noise frequency ranges. Error bars: ±SEM. ∗ for technical reasons only 4 subjects could be measured with DPOAEs in the OBN-16 kHz and in the ACN + OBN-16 kHz groups.

Figure 2 shows DP-grams obtained 4 weeks post-exposure in rats subjected to (a) 4, (b) 8, or (c) 16-kHz OBNs with or without co-exposure to ACN. For reference, DP-grams obtained in controls and ACN alone are included in each of the graphs.

Equivalent baseline DP grams were obtained in the different groups prior to exposure (not shown).

Four weeks post-exposure, the global 2-way ANOVA reveals significant effects of “exposure” on the DPOAE amplitudes (*F*(7, 44) = 5.955; *P* < .0001). The results of the post-hoc Bonferroni's tests are detailed in what follows.

Figure 2(a) displays the DPgrams obtained 4 weeks after exposure to the 110 dB OBN-4 kHz noise with or without ACN. Exposure to noise alone only induced a slight DPOAE decrease from about 6 kHz to the lower limit of the “notch.” Above this notch the decrease appears more profound. The difference from controls is only significant from 36 to 55 kHz by Bonferroni's test (Figure 5(a)). The animals that were exposed to ACN and noise show lower DPOAEs than the noise-alone exposed rats over a wide range of frequencies. However, this difference is only significant from 16 to 28 kHz (*P* < .05), while it approaches significance from 9 to 15 kHz. As noted earlier, 4 frequencies (20.8, 22.2, 23.8, and 25.6 kHz were excluded from this analysis. ACN-alone animals do not show any significant decrease of their DPOAE amplitudes when compared to control animals.

Figure 2(b) displays the DPgrams obtained 4 weeks after exposure to the 97-dB OBN-8 kHz noise with or without ACN. The exposure to ACN + OBN-8 kHz (Figure 2(b)) induced a large potentiation of NIHL. The region in which the mean differences were greatest cannot be precisely determined due to the notch in the responses, but it can be estimated that the difference between ACN + OBN-8 kHz and the noise-alone animals is about 18–25 dB between 17 and 36 kHz. Bonferroni's post-test (Figure 5(a)) reveals that the ACN + noise animal's DPOAE amplitudes differ from the ones obtained in noise-alone animals from 15 to 55 kHz; while the animals exposed to noise alone are not different from controls.

In a similar fashion, the 97-dB OBN-16 kHz exposure (Figure 2(c)) did not induce any permanent decrease of DPOAE amplitudes, while the ACN + OBN-16 kHz exposure caused a reliable DPOAE deficit above 12 kHz. The mean difference was the greatest between 27 and 37 kHz where it reached more than 30 dB. The only significant difference shown by Bonferroni's post-tests is between noise-alone and ACN + noise DPgrams between 12 and 45 kHz (*P* < .05).

#### 3.1.2. Compound Action Potentials

**Figure 3:**
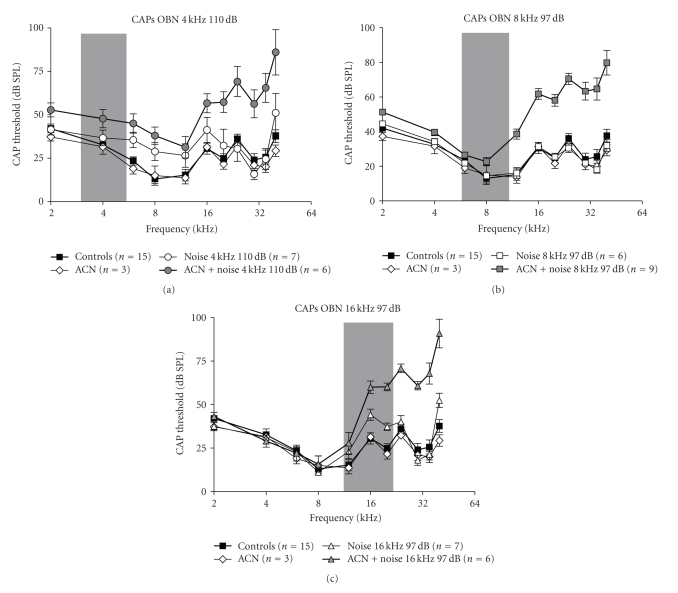
Effects of the different experimental treatments on compound action potential (CAP) thresholds measured 4 weeks post-exposure for frequencies ranging from 2 to 40 kHz. CAP thresholds obtained in rats exposed to (a) the 110 dB OBN-4 kHz with or without ACN, (b) to the 97 dB OBN-8 kHz with or without ACN, or (c) to the 97 dB OBN-16 kHz with or without ACN (see Table 1  for details). Auditory thresholds measured in control and ACN alone animals were included in each of the graphs for reference. The colored areas represent the octave band-noise frequency ranges. Error bars: ± SEM.

Figure 3 presents the disruption of CAP thresholds measured 4 week post-exposure in rats subjected to (a) 4, (b) 8, or (c) 16-kHz OBN with or without co-exposure to ACN. For reference, thresholds obtained in controls and ACN alone are included in each of the graphs.

The two-way ANOVA carried out on the CAP thresholds revealed a significant effect of “exposure” (*F*(7, 49) = 13.16; *P* < .0001). ACN alone animals did not show any significant threshold difference compared to the control thresholds (Bonferroni, *P* > .05; see Figure 5(b)). While the OBN-4 kHz animals show slightly elevated thresholds between 6 and 16 kHz compared to the control data, this increase is not statistically significant (*P* > .05). By contrast, the animals that received combined exposures to ACN + OBN-4 kHz display significantly elevated thresholds between 20 and 40 kHz when compared to the noise-alone thresholds (*P* < .05; see Figure 5(b)). The difference between these two groups reaches up to 41 dB at both 30 and 35 kHz.

Neither the 8-kHz nor the 16-kHz noise-alone groups show significantly elevated thresholds compared to controls (Bonferroni, *P* > .05). By contrast, the ACN + OBN-8 kHz and the ACN + OBN-16 kHz thresholds were significantly different from their noise-alone counterparts, respectively, between 12 and 40 kHz and between 20 and 40 kHz (Figure 5(b)).

### 3.2. Hair Cell Counts

To assess the magnitude of cochlear damage, hair cells were counted from cochleae harvested from the same animals used for physiological studies. The hair cell loss is presented as cochleograms that display the percentage of hair cell loss as a function of distance from the apex of the cochlea.

**Figure 4:**
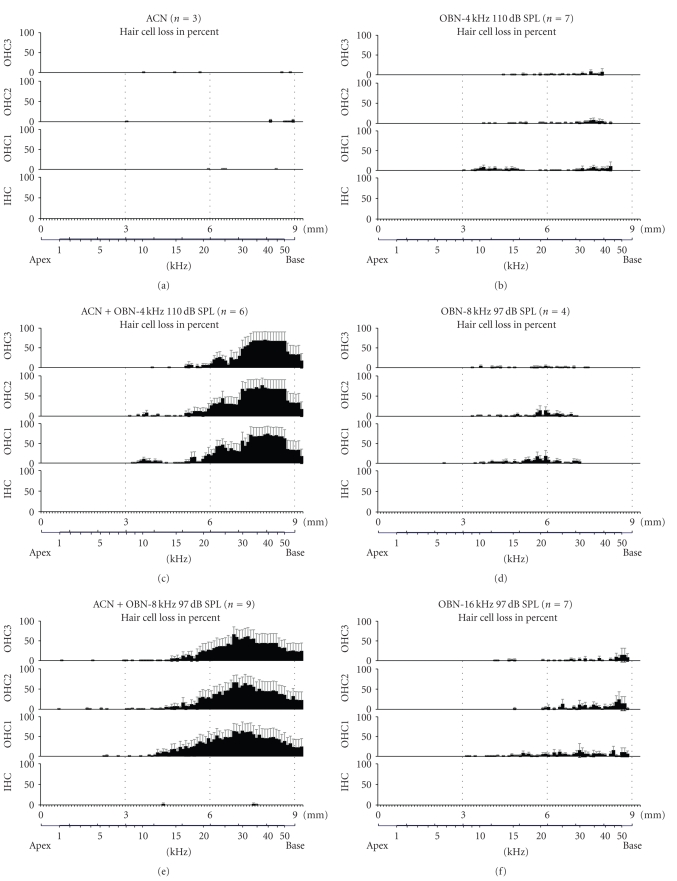

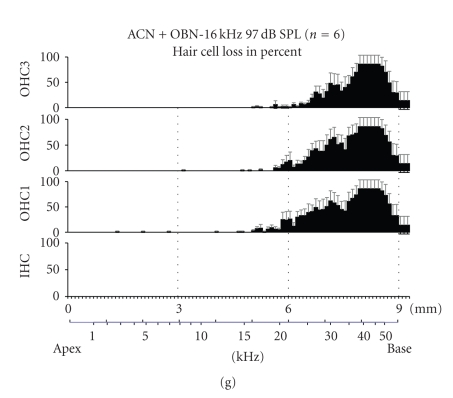
Average cochleograms showing hair cell loss in rats exposed to (a) ACN alone or to the 110 dB OBN-4 kHz with (c) or without (b) ACN, in rats exposed to the 97 dB OBN-8 kHz with (e) or without (d) ACN, and in rats exposed to the 97 dB OBN-16 kHz with (g) or without (f) ACN. Abscissa (i) upper trace: length (mm) of the entire spiral course of the organ of Corti from the bottom of the hook, (ii) lower trace: frequency-map according to Muller [9]. Ordinate: hair cell loss in percent. IHC: inner hair cells; OHC1: first row of outer hair cells; OHC2: second row; OHC3: third row. Error bars represent the standard error.

Figure 4 shows the mean cochleograms obtained in the animals exposed to (a) ACN alone, (b) OBN-4 kHz alone, (c) ACN + OBN-4 kHz, (d) OBN-8 kHz alone, (e) ACN + OBN-8 kHz, (f) OBN-16 kHz alone, (g) ACN + OBN-16 kHz. The cochleae from controls (not shown) and ACN alone subjects (Figure 4(a)) displayed no hair cell loss, while those exposed to any of the OBNs without ACN (Figures 4(b), 4(d)  and 4(f)) displayed limited damage in the basal half of the organ of Corti, less than 10% OHC loss at any tonotopic location. None of the exposures yielded any inner hair cell (IHC) loss. Therefore, IHCs are not discussed here, and are not included in the statistical analysis (see Section 2.8).

Consistent with physiological results, the cochleae from rats exposed to both ACN and noise (Figures 4(c), 4(e), and 4(g)) exhibited substantial damage in the basal (or high frequency) third of the organ of Corti. The pattern of OHC loss is actually surprisingly similar among the different ACN + noise exposures. Despite the fact that the OHC loss obtained in the rats exposed to ACN + OBN-8 kHz is scattered over a wider range than the other two combined exposure groups, the hair cell loss averaged about 45% in the three OHC rows in the region corresponding to frequencies above 15 kHz for the three different combined exposures.

**Figure 5:**
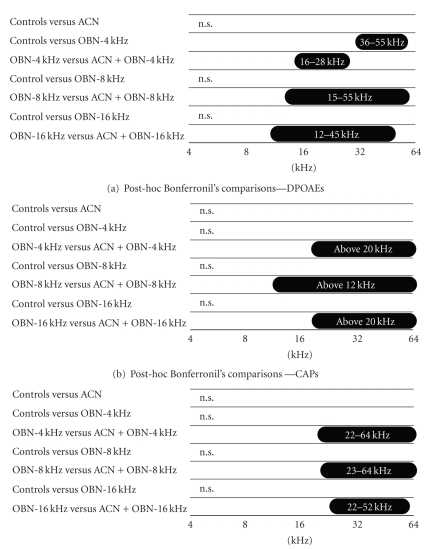
Statistical comparisons between groups for (a) DPOAE amplitudes, (b) CAP thresholds, and (c) hair cell loss at 4 weeks postexposure: Bonferroni's post-hoc comparisons with *P* = .05 as the significant limit. The parent analyses were repeated measure ANOVAs with “exposure” as between factor and “frequency” as a within factor. The colored bands illustrate the frequency range for which Bonferroni's comparisons between groups yielded *P* < .05. ns: not significantly different. *Note: for the CAP technique, the significance range is presented as “above x kHz” because it does not allow measurements above 40 kHz, whereas the significance is likely more extended toward high frequencies*.

The OHC loss obtained in all experimental groups was analyzed with a global two-way ANOVA. The overall effect of “exposure” was significant (*F*(7, 47) = 5.60;  *P* = .0003). Bonferroni's post-tests (see graphic representation in Figure 5(c)) confirmed that cochleograms obtained with either one of the OBNs were not significantly different from the control cochleogram (not shown). By contrast, the ACN + OBN-4 kHz, the ACN + OBN-8 kHz, and the ACN + OBN-16 kHz cochleograms are significantly different from their noise-alone counterparts, respectively, from 22 to 64 kHz, from 23 to 64 and from 26 to 52 kHz (*P* < .05). Hair cell loss in the ACN + OBN-8 kHz was visibly wider in terms of frequency than the OHC loss obtained in the other two combined exposure groups. However, the difference barely reached statistical significance at a few frequencies: OHC loss in the ACN + OBN-8 kHz cochleae was different (*P* < .05) from the loss in the ACN + OBN-4 kHz at 27, 28, 37–39, and 46–50 kHz, and from the ACN + OBN-16 kHz cochleae at 21, 22–25, 27-28, and 38–42 kHz.

## 4. Discussion

This study was carried out to investigate the relationship between the frequency range of continuous OBN exposures and the tonotopic location of the cochlear damage with and without co-exposure to ACN, a compound that decreases cellular antioxidant defenses. The results show that, when ACN is present, moderate noise exposures centered at 4, 8, and 16 kHz all yield high frequency hearing impairment and basal OHC damage. In fact, basal hair cell loss seems nearly independent of the noise frequency band used in exposures. By contrast, without ACN treatment, the damage induced by the different noise exposures were very limited. Also, exposure to ACN alone did not cause permanent DPOAE decrease, CAP threshold shift, or loss of hair cells.

Despite some differences in the pattern of the OHC loss, the maximal damage caused by ACN + noise exposures is always located around 30–40 kHz, regardless of the center frequency of the OBN. This observation underscores the fact that the noise exposures used in this investigation include cochlear effects that are not limited to the frequency ranges of the OBNs, but extend to the basal end of the cochlea.

Histological observations confirm that up to a 3-octave shift exists between the OBN center frequency and the corresponding frequency sensitivity of the area of maximal cochlear damage. Exposure to noise-alone caused minimal cochlear damage located within one octave above the OBN (Figures 4(b), 4(d), and 4(f)), which confirms that the losses at high frequencies are a consequence of the combined ACN + noise effect.

Our results are consistent with those obtained by Chen and Fechter [10] who showed by exposing rats to carbon monoxide and different OBNs that the potentiation was much bigger for the high frequency exposures. Also, Rao and Fechter [11] demonstrated that phenyl-N-tert-butylnitrone (PBN), a spin-trap agent which neutralizes ROS, significantly protected the cochlea against the interactive effect of CO + noise. This protection was, again, only significant at high frequencies. The fact that we observed similar high frequency potentiation might suggest that ACN and CO share common interactions with the cochlear defenses against NIHL.

While ACN does not impair oxygen delivery, it directly decreases tissue antioxidant defenses [12–15]. Further, injection of 50 mg/kg ACN in the rat depletes liver and cochlear GSH by 60–80% within 30 minutes [5]. The potentiation of NIHL (OBN-8 kHz, 97 dB SPL, 5 × 4 hours) by this ACN injection could be reduced by a pre-treatment with L-N-acetylcysteine, a compound that increases cellular GSH levels. This treatment was especially effective for the frequencies above 30 kHz.

Several other studies offer a solid basis to hypothesize that the shift of the impairment towards high frequencies may be due to the fact that the cochlea has different susceptibilities to ROS depending on the tonotopic location. In vitro , Sha et al. [16] observed that cochlear organotypic cultures of basal OHCs were more vulnerable to free-radical damage than apical OHCs, and that basal OHC survival was improved by the addition of L-NAC or GSH. In accordance with this result, Clerici and Yang [17] showed in vivo that direct perilymphatic generation of ROS (by instillation of artificial supplemented perilymph) induced a rapid degradation in high-frequency CAP threshold sensitivity, the pattern of which being surprisingly similar to the threshold shift obtained in our ACN + noise exposed animals (Figure 3). The literature thus suggests that the decrease of antioxidant defenses caused by ACN renders the basal OHCs more susceptible to noise than the apical OHCs. This differential vulnerability gives a possible explanation for the high-frequency shift phenomenon observed in our experiments.

In the present study, two different audiometric techniques were used in order to estimate both the global auditory sensitivity (CAPs) and the physiological state of the OHC stereociliae (DPOAEs). As a general rule, data obtained with these two techniques were in excellent agreement with the histology. However, in terms of frequency range, CAPs threshold shifts were more closely related to the OHC loss than to the DPOAEs (Figure 5). The DPOAEs were especially inconsistent with the histology for the ACN + OBN-4 kHz exposure (Figure 2(a) versus Figure 4(c) and Figure 5(a) versus. Figure 5(c)). The potentiation appeared significant between 16 and 29 kHz with DPOAEs and between 22 and 64 kHz for the OHC loss. We suggest that this discrepancy is likely due to the fact that DPOAE decrements are better explained in terms of OHC dysfunction, rather than actual OHC death. The DPOAE patterns obtained in the animals exposed to the lower noise band suggest that this exposure was not traumatic enough to kill the hair cells although it did alter OHC function.

## Figures and Tables

**Table 1:**
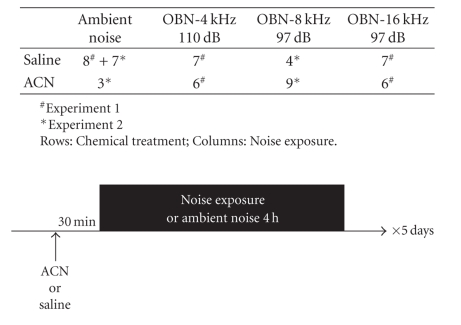
Experimental groups and treatment schedules.

## Abbreviations:

NIHL: Noise-induced hearing loss
OBN: Octave band noise
ACN: Acrylonitrile
OHC: Outer hair cell
GSH: Glutathione
ROS: Reactive oxygen species
DPOAE: Distortion Product Otoacoustic Emission
CAP: Compound Action Potential.
